# Mapping endemic freshwater fish richness to identify high‐priority areas for conservation: An ecoregion approach

**DOI:** 10.1002/ece3.10970

**Published:** 2024-02-16

**Authors:** Masoud Yousefi, Arash Jouladeh‐Roudbar, Anooshe Kafash

**Affiliations:** ^1^ Stiftung Neanderthal Museum Mettmann Germany; ^2^ Leibniz Institute for the Analysis of Biodiversity Change (LIB) Museum Koenig Bonn Germany; ^3^ Department of Fisheries University of Tehran Tehran Iran; ^4^ School of Culture and Society Aarhus University Aarhus C Denmark

**Keywords:** conservation, freshwater ecosystem, last glacial maximum, past climate, richness mapping, Zagros Mountains

## Abstract

Freshwater ecosystems are experiencing accelerating global biodiversity loss. Thus, knowing where these unique ecosystems' species richness reaches a peak can facilitate their conservation planning. By hosting more than 290 freshwater fishes, Iran is a major freshwater fish hotspot in the Middle East. Considering the accelerating rate of biodiversity loss, there is an urgent need to identify species‐rich areas and understand the mechanisms driving biodiversity distribution. In this study, we gathered distribution records of all endemic freshwater fishes of Iran (85 species) to develop their richness map and determine the most critical drivers of their richness patterns from an ecoregion approach. We performed a generalized linear model (GLM) with quasi‐Poisson distribution to identify contemporary and historical determinants of endemic freshwater fish richness. We also quantified endemic fish similarity among the 15 freshwater ecoregions of Iran. Results showed that endemic freshwater fish richness is highest in the Zagros Mountains while a moderate level of richness was observed between Zagros and Alborz Mountains. High, moderate, and low richness of endemic freshwater fish match with Upper Tigris & Euphrates, Namak, and Kavir & Lut Deserts ecoregions respectively. Kura – South Caspian Drainages and Caspian Highlands were the most similar ecoregions and Orumiyeh was the most unique ecoregion according to endemic fish presence. Precipitation and precipitation change velocity since the Last Glacial Maximum were the most important predictors of endemic freshwater fish richness. Areas identified to have the highest species richness have high priority for the conservation of freshwater fish in Iran, therefore, should be considered in future protected areas development.

## Graphical abstract

Iran's freshwater ecosystems are home to 85 endemic fish species. By analyzing distribution records and using a generalized linear model, the researchers identified the Zagros Mountains as having the highest endemic fish richness, followed by a moderate level between the Zagros and Alborz Mountains. They found that precipitation and precipitation change since the Last Glacial Maximum were the key factors influencing fish richness. These findings highlight the importance of prioritizing conservation efforts in the identified areas of high species richness for the protection of Iran's freshwater fish.

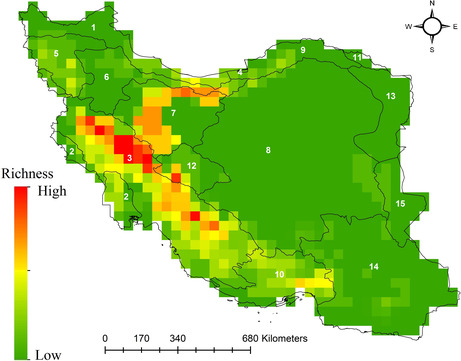

## 1 INTRODUCTION

Freshwater ecosystems (rivers, lakes, and wetlands) support one‐third of global vertebrates despite covering 1% of the Earth's surface (Balian et al., 2008; Gleick, 1998; Strayer & Dudgeon, 2010). They provide essential ecosystem services and benefits like drinking water, recreation and tourism, water for agriculture and energy, habitat for threatened species, and microclimate regulation (Chopra et al., 2005; Hanna et al., 2018; Kaval, 2019; Sun et al., 2017; Vári et al., 2022). But climate change, land use change, habitat destruction, dam building, pollution, and invasive species are causing significant negative impacts on freshwater ecosystems worldwide (Arthington et al., 2016; Boyero et al., 2012; Chopra et al., 2005; Cianfrani et al., 2018; Collen et al., 2014; Dudgeon et al., 2006; Strayer & Dudgeon, 2010; Dudgeon, 2011; WWF, 2020). A key concern of conservation biology is to conserve freshwater ecosystems since they face multiple threats (Maasri et al., 2022; Vári et al., 2022; WWF, 2020). Therefore, understanding freshwater species richness and spatial distribution is of utmost importance for identifying priority areas for their conservation. Although spatial distributions of invertebrates have been extensively studied in freshwater ecosystems (Downing & Leibold, 2010; Hamada et al., 2002; Jacobsen, 2004; Miller et al., 2018; Sickle et al., 2006; Wiberg‐Larsen et al., 2000) little research has been done on vertebrates of freshwater ecosystems (Cianfrani et al., 2018; Collen et al., 2014; Niu et al., 2012; Uchida & Inoue, 2010).

With more than 18,000 species, freshwater fishes are among the most diverse taxonomic groups and are known for a high degree of endemism (Helfman et al., 2009; Hughes, 2021; van der Sleen & Albert, 2022). In fact, 51% of all 35,768 known fish species live in freshwater ecosystems (Hughes, 2021). Due to their dispersal limitations and rapid decline in freshwater habitats, freshwater fishes are one of the most threatened groups of animals (Arthington et al., 2016; Dawson, 2012; Dudgeon et al., 2006; Dudgeon, 2011; Gozlan et al., 2019; Vári et al., 2022). They face threats such as river regulation, dam construction, land use change, hydropower development, habitat fragmentation, climate change, and invasive species (Arthington et al., 2016; Dawson, 2012; Dudgeon et al., 2006; Szabolcs et al., 2022). A recent study has shown that one‐third of freshwater fishes are threatened with extinction (Hughes, 2021). This group is less studied compared to terrestrial vertebrate groups like birds, mammals, and reptiles (Grenyer et al., 2006; Jenkins et al., 2013; Rahbek & Graves, 2001; Roll et al., 2017). Thus, knowing where their richness is highest is essential for identifying high‐priority areas for conservation.

Iran hosts high‐species diversity and endemism due to its rich geological history, several phases of glacial and interglacial, sea level fluctuations and the presence of several major geographical barriers (Reviewed in Yousefi et al., 2023). The country is home to more than 290 freshwater fishes belonging to 102 genera and 33 families (Jouladeh‐Roudbar et al., 2020). Historical events like mountain uplifting and past climatic fluctuations are known as important drivers of freshwater fish distribution and speciation in Iran (Esmaeili, Teimori, Gholami, & Reichenbacher, 2014, Esmaeili, Teimori, Sayyadzadeh, et al., 2014; Ghanavi et al., 2016; Gholami et al., 2014; Schwarzer et al., 2017; Zarei et al., 2021; Zareian et al., 2018). For instance, Schwarzer et al. (2017) suggested several scenarios to explain genetic divergence and phenotypic differentiation between geographical populations of *Iranocichla* in Iran due to past climatic fluctuations. Thus, historical factors might be the most important drivers of current distribution pattern of freshwater fishes in Iran.

Freshwater ecosystems of Iran are experiencing accelerating biodiversity loss due to the growing human activities. These ecosystems are facing important threats like climate change, land use change, invasive species, and habitat fragmentation through dam construction unregulated water absorption, habitat destruction, domestic and industrial pollution, agricultural runoff, and overfishing. Thus, it is necessary to identify species‐rich areas for conservation planning in the country. The aims of this study are to create the first richness map of endemic freshwater fishes of Iran and explore the drivers of their richness pattern. This study also aimed to document the distribution and similarity of endemic freshwater fishes within the freshwater ecoregions of Iran. Considering that freshwater fishes have low dispersal ability (less likely that responded to contemporary factors) and that previous studies have indicated that past climate change played an important role in shaping vertebrate distribution in Iran (Bartáková et al., 2015; De Bie et al., 2012; Shurin et al., 2009; Yousefi et al., 2023), we hypothesize that past climate is more influential factor in shaping freshwater fish richness in Iran. We are expecting to find a negative association between fish richness and high climate change in the past (García‐Andrade et al., 2021; Gavin et al., 2014; Holderegger & Thiel‐Egenter, 2009; Jansson, 2003; Sandel et al., 2011; Tedesco et al., 2005); on the contrary, areas which experienced higher climatic changes in the past should host lower species diversity compared to regions which experienced non or little climatic changes (Brown et al., 2020; Harrison & Noss, 2017; Jansson, 2003; Sandel et al., 2011).

## 2 MATERIALS AND METHODS

### 2.1 Study area

**FIGURE 1:**
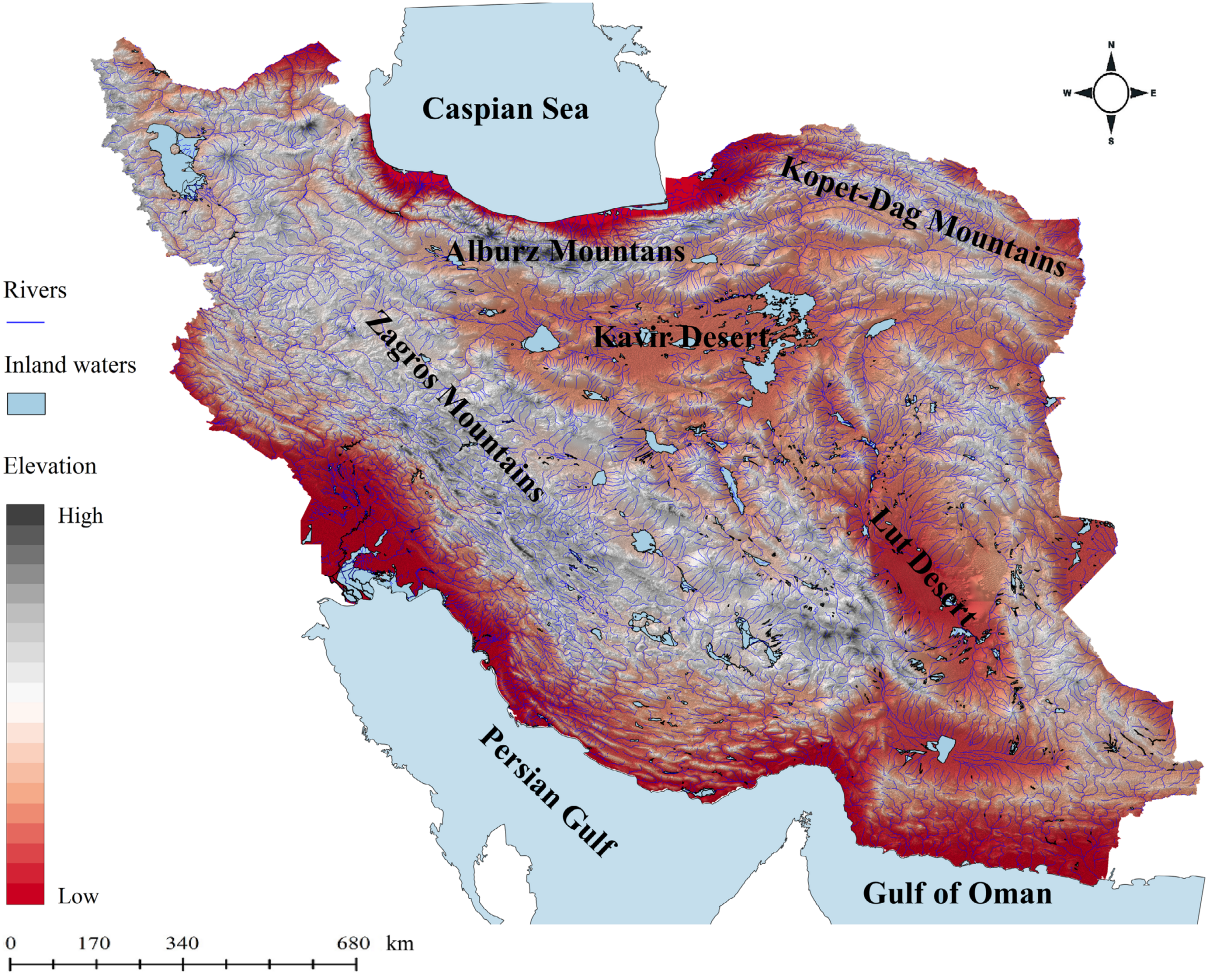
Iran's topographic map (Jarvis et al., 2008) with its major topographic features, rivers, and inland waters.

Iran with an area of about 165 million hectares stands as the second‐largest country in Western Asia. The country has a predominantly arid to semi‐arid climate, with more than 80% of its land receiving <250 mm of annual rainfall. The elevation ranges from 26 to 5671 m in the country. The Alborz, Zagros, and Kopet Dagh mountains are the major mountain chains in the country (Figure 1). The Alburz Mountains in northern Iran stretch from west to east at the southern coast of the Caspian Sea and form the northern border of the Iranian Plateau (Kafash et al., 2020). The Kopet Dagh Mountains is a large mountain range which is located in the northeast of Iran between Iran and Turkmenistan. The Zagros Mountains form the western and south‐western borders of the Iranian Plateau, covering 1500 km from Lake Van in Turkish Kurdistan to south‐eastern Iran (Kafash et al., 2020; Yousefi et al., 2023). The freshwater fish fauna of Iran draws significant influence from the Palearctic, Afrotropical, and Indomalayan biogeographic regions (Jouladeh‐Roudbar et al., 2020).

### 2.2 Endemic freshwater fish checklist and distribution records

**FIGURE 2:**
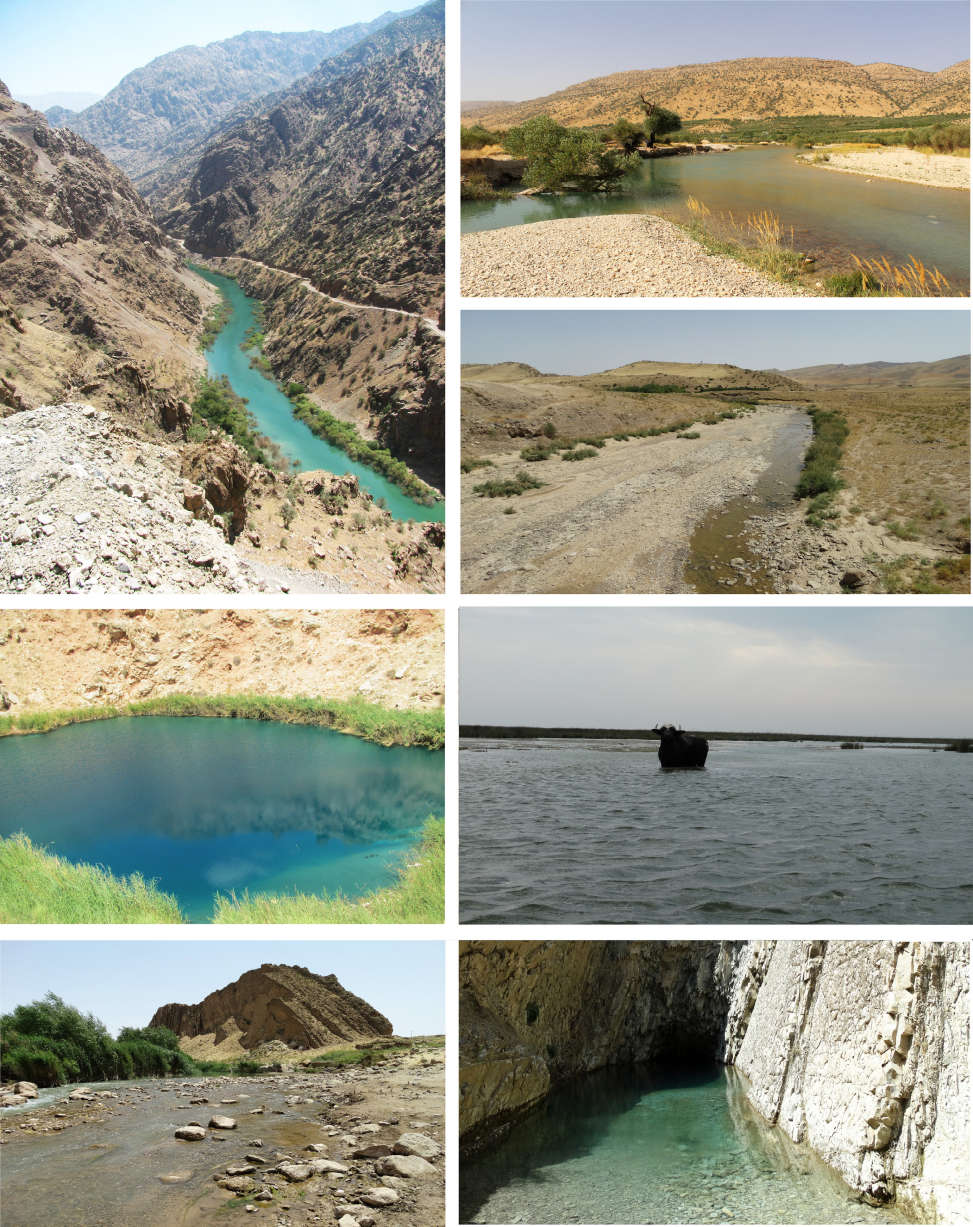
Photos of some surveyed habitats of endemic freshwater fishes of Iran. From top left Sivan River; Siyah gav Lake; Hari River, Beshar River; Kashaf River; Shadegan wetland; Loen cave. Photos by Arash Jouladeh‐Roudbar.

To map endemic freshwater fish richness in Iran, we first prepared a checklist of endemic freshwater fishes for the country based on the available published checklists (Jouladeh‐Roudbar et al., 2020). This checklist contains all valid endemic freshwater fishes of Iran until December 2022 (Table 1). In this study, distribution records of the endemic freshwater fish are based on our team's long‐term fieldworks (Figure 2) and opportunistic observations of freshwater fishes in Iran (Kafash, 2023; Yousefi et al., 2024). Fish sampling activities spanned across all Iranian basins from 2005 to 2022 (Jouladeh‐Roudbar et al., 2015, 2017, 2020), employing a range of methods including the backpack electro‐fisher (Samus 1000), hand nets, gill nets, and casting nets. Upon collection, fish specimens were meticulously identified at the collection sites, facilitated by a diagnostic key developed by Jouladeh‐Roudbar et al. (2020). Following identification and thorough recording of data, the fish were thoughtfully released back into their natural habitats, ensuring minimal disruption to their ecosystems. Sampling was conducted from sunrise to sunset during the sampling days. It is important to note that since we recorded each species' geographic location (Appendix S1) with high accuracy using the Garmin GPS device (GRGPSMAP65S), our data is not associated with identification and spatial errors (Yousefi et al., 2020).

**TABLE 1:** Checklist of endemic freshwater fishes of Iran.

Order	Family	Species
Cypriniformes	Cyprinidae	*Barbus karunensis*

*Barbus miliaris*	

*Capoeta aculeata*	

*Capoeta alborzensis*	

*Capoeta anamisensis*	

*Capoeta buhsei*	

*Capoeta coadi*	

*Capoeta ferdowsii*	

*Capoeta gracilis*	

*Capoeta mandica*	

*Capoeta pyragyi*	

*Capoeta saadii*	

*Capoeta shajariani*	

*Carasobarbus sublimus*

*Cyprinion tenuiradius*

*Garra gymnothorax*

*Garra hormuzensis*

*Garra lorestanensis*

*Garra mondica*

*Garra nudiventris*

*Garra persica*

*Garra roseae*

*Garra tashanensis*

*Garra tiam*

*Garra typhlops*

Gobionidae	*Romanogobio persus*

Leuciscidae	*Acanthobrama persidis*

	*Acanthobrama urmianus*

	*Alburnoides damghani*

	*Alburnoides idignensis*

	*Alburnoides namaki*

	*Alburnoides nicolausi*

	*Alburnoides qanati*

*Alburnoides samiii*

*Alburnoides tabarestanensis*

*Alburnus doriae*

*Chondrostoma esmaeilii*

*Chondrostoma orientale*

*Squalius namak*

Cobitidae	*Cobitis avicennae*

	*Cobitis faridpaki*

*Cobitis linea*

Nemacheilidae	*Eidinemacheilus smithi*

	*Oxynoemacheilus karunensis*

	*Oxynoemacheilus kiabii*

	*Oxynoemacheilus marunensis*

	*Oxynoemacheilus persa*

	*Oxynoemacheilus tongiorgii*

	*Paracobitis abrishamchianorum*

	*Paracobitis basharensis*

	*Paracobitis hircanica*

	*Paracobitis malapterura*

	*Paracobitis persa*

	*Paraschistura abdolii*

	*Paraschistura aredvii*

*Paraschistura delvarii*

*Paraschistura hormuzensis*

*Paraschistura ilamensis*

*Paraschistura kermanensis*

*Paraschistura makranensis*

*Paraschistura naumanni*

*Paraschistura nielseni*

*Paraschistura susiani*

*Sasanidus kermanshahensis*

*Turcinoemacheilus bahaii*

*Turcinoemacheilus hafezi*

*Turcinoemacheilus saadii*
Siluriformes	Bagridae	*Mystus cyrusi*
	Sisoridae	*Glyptothorax alidaeii*
		*Glyptothorax galaxias*
		*Glyptothorax hosseinpanahii*
		*Glyptothorax shapuri*
		*Glyptothorax silviae*
Gobiiformes	Gobiidae	*Ponticola hircaniaensis*
		*Ponticola iranicus*
		*Ponticola patimari*
Cichliformes	Cichlidae	*Iranocichla hormuzensis*
Cyprinodontiformes	Aphaniidae	*Aphaniops furcatus*
		*Aphaniops ginaonis*
		*Aphaniops teimorii*
		*Esmaeilius isfahanensis*
		*Esmaeilius darabensis*
		*Esmaeilius persicus*
		*Esmaeilius shirini*
		*Esmaeilius vladykovi*

### 2.3 Environmental and historical predictors

Since the goal of this research is to disentangle the role of historical climatic variables vs. contemporary climatic variables in shaping the current richness pattern of endemic freshwater fish species in Iran, we included variables (Table 2) that are known to be important in shaping biodiversity distribution patterns in Iran (Hosseinzadeh et al., 2014; Kafash et al., 2020, 2021; Yousefi et al., 2022, 2023). For the current climate, we took into account annual mean temperature and annual mean precipitation, which are freely accessible from WorldClim (Fick & Hijmans, 2017). To explore the role of Pleistocene climate fluctuations in shaping endemic freshwater fish richness we considered climate change velocity which is a measurement for long‐time climate variability and it shows the direction and rate at which organisms must have moved to maintain a given climate under climate change (Sandel et al., 2011). We estimated changes in climate between the Last Interglacial (LIG ~130,000 BP) and the Last Glacial Maximum (LGM ~21,000 BP) today as well as the LGM and current climate (Sandel et al., 2011) in VoCC r package (García Molinos et al., 2019). Please check García Molinos et al. (2019) to see details of climate change velocity estimation. The LIG and LGM climate data were downloaded from PaleoClim which is a source of free, high‐resolution paleoclimate data for biological modeling and GIS (Brown et al., 2018).

**TABLE 2:** List of predictors used to explore drivers of freshwater fish richness in Iran.

Predictor	References
Annual mean temperature	Fick and Hijmans (2017)
Annual mean precipitation	Fick and Hijmans (2017)
Temperature change velocity (LGM)	Brown et al. (2018)
Precipitation change velocity (LGM)	Brown et al. (2018)
Temperature change velocity (LIG)	Brown et al. (2018)
Precipitation change velocity (LIG)	Brown et al. (2018)

### 2.4 Richness mapping and statistical analysis

We mapped the 85 endemic fish species with the outline method, Minimum Convex Polygons, in the adehabitatHR package (Calenge, 2011). Then we summed all created maps to produce an endemic freshwater fish richness map in the raster package (Hijmans & Van Etten, 2021). We fitted a generalized linear model (GLM) with quasi‐Poisson distribution to determine the relationship between endemic freshwater fish richness and the historical and contemporary variables. We computed the explained deviance for each variable separately and in combinations to identify the most crucial driver of endemic freshwater fish richness using the “ecospat.adj.D2.glm” function in the R‐package “ecospat” (Di Cola et al., 2017) in R environment (R Core Team, 2017).

### 2.5 The similarity of endemic fish assemblages among the freshwater ecoregions

**FIGURE 3:**
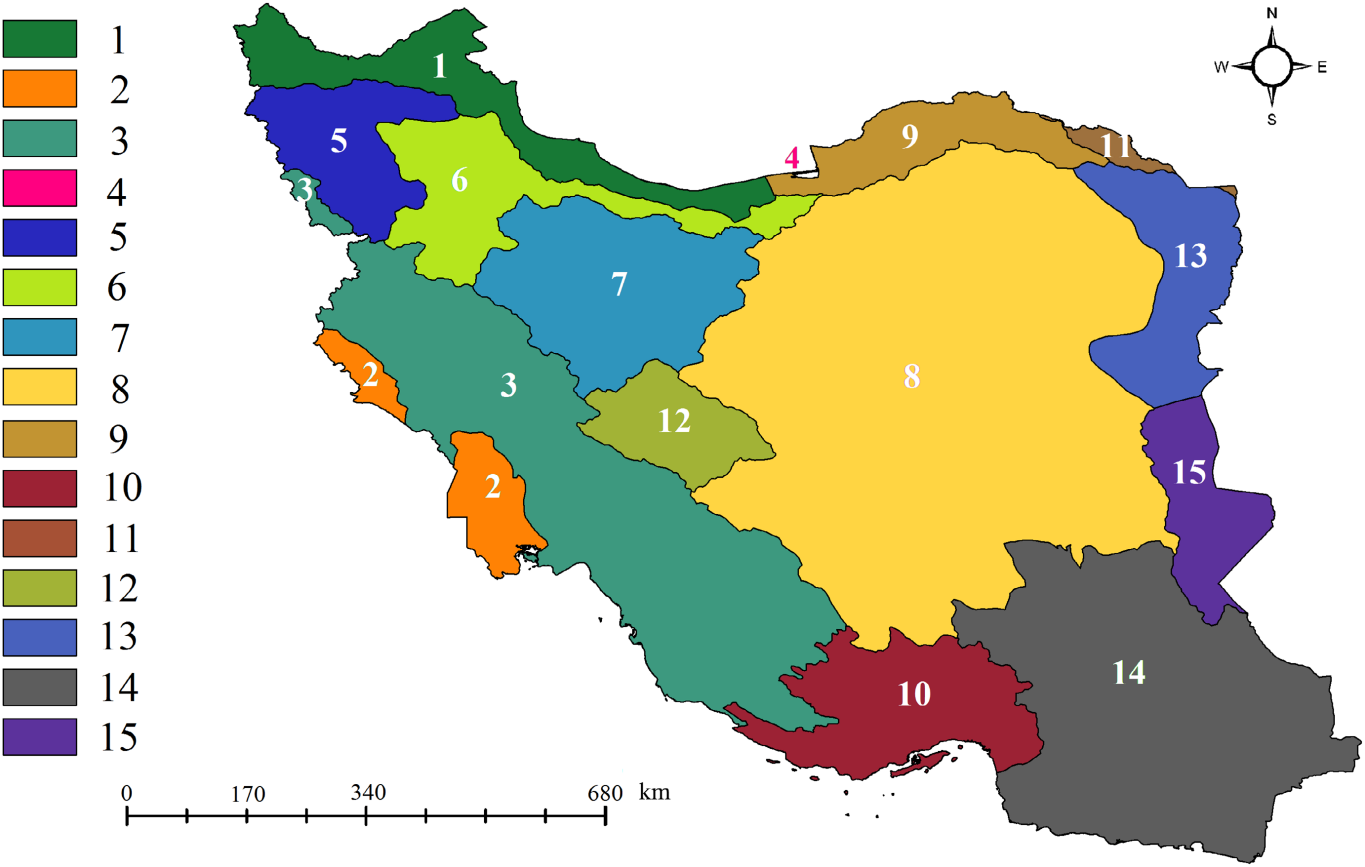
Freshwater ecoregions of Iran (Abell et al., 2008). 1. Kura – South Caspian Drainages, 2. Lower Tigris & Euphrates, 3. Upper Tigris & Euphrates, 4. Caspian Marine, 5. Orumiyeh, 6. Caspian Highlands, 7. Namak, 8. Kavir & Lut Deserts, 9. Turan Plain, 10. Northern Hormuz Drainages, 11. Middle Amu Darya, 12. Esfahan, 13. Upper Amu Darya, 14. Baluchistan, 15. Helmand – Sistan.

In the present research, we quantified endemic freshwater fish distribution and similarity among the 15 freshwater ecoregions of Iran based on the presence records of the 85 endemic fish species (Table 1). In most cases of freshwater diversity mapping studies, fish species were analyzed based on watersheds (Anas & Mandrak, 2021; García‐Andrade et al., 2021; Miller & Román‐Palacios, 2021; Oberdoff et al., 1995, Oberdorff et al., 1997, 2011; Qian et al., 2021; Tedesco et al., 2017) but here we applied an ecoregion approach. To assess the similarity of endemic fish assemblages among the ecoregions (Figure 3), we created a presence–absence matrix of all endemic fish species within the above‐mentioned ecoregions. Then we used the Jaccard similarity index in the Past software (Hammer et al., 2001) to estimate the similarity of endemic freshwater fish assemblages among the ecoregions.

## 3 RESULTS

### 3.1 Richness map

**FIGURE 4:**
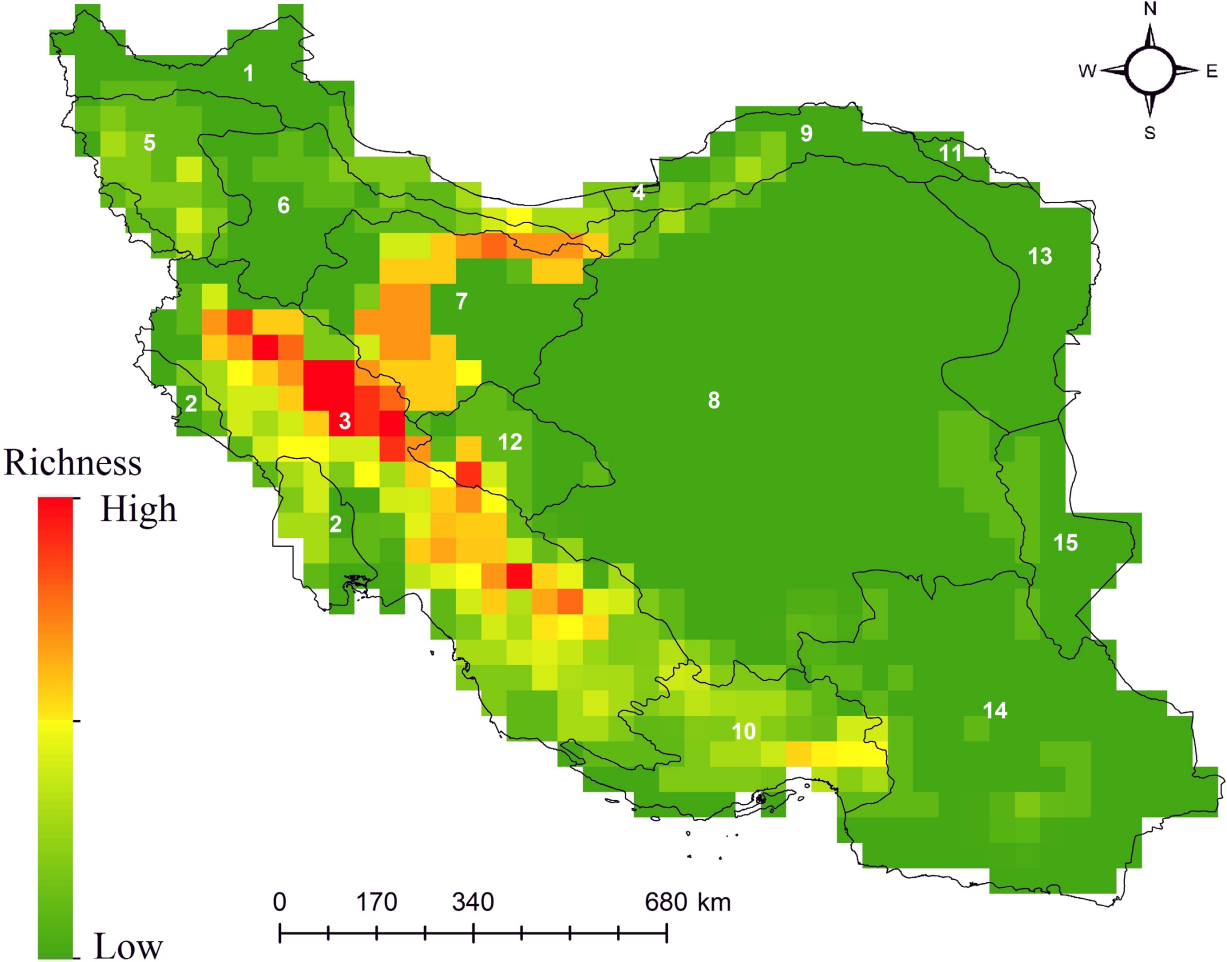
Richness map of 85 endemic freshwater fishes of Iran. Species richness ranges from 0 in Middle Amu Darya and Upper Amu Darya ecoregions to 13 in Upper Tigris & Euphrates. Polygons and numbers show Iran's freshwater ecoregions as follows: 1. Kura – South Caspian Drainages, 2. Lower Tigris & Euphrates, 3. Upper Tigris & Euphrates, 4. Caspian Marine, 5. Orumiyeh, 6. Caspian Highlands, 7. Namak, 8. Kavir & Lut Deserts, 9. Turan Plain, 10. Northern Hormuz Drainages, 11. Middle Amu Darya, 12. Esfahan, 13. Upper Amu Darya, 14. Baluchistan, 15. Helmand—Sistan.

Until now, 85 endemic freshwater fishes were described from Iran. Family Nemacheilidae and Cyprinidae were the most diverse family each with 25 endemic species. *Capoeta*, with 11 species and *Garra* and *Paraschistura*, each with 10 species were the most spacious genera. A richness map was developed based on all 85 fish species distribution maps. Results showed that endemic freshwater fish richness is highest in the Zagros Mountains and moderate level of richness was observed between Zagros and Alborz Mountains. High, moderate, and low richness of endemic freshwater fish match with Upper Tigris & Euphrates, Namak, and Kavir & Lut Deserts ecoregions, respectively (Figure 4). The Number of endemic freshwater fish ranges from 0 in central Iran to 13 in the Zagros Mountains (Figure 4).

### 3.2 Variable importance

Precipitation was identified as the most influential determinant of endemic freshwater fish richness, with a significant positive correlation (*p* < .000), meaning that areas with higher precipitation host more species. Precipitation explained the highest proportion of variance (37.5%) among all historical and contemporary variables. Precipitation change velocity since the LGM was the second most important predictor (24.5%) of endemic freshwater fish richness. The correlation of precipitation change velocity with richness was negative (*p* < .000), which meant that areas with rapid precipitation changes hosted fewer species (Table 3).

**TABLE 3:** Results of generalized linear model with quasi‐Poisson distribution to identify the most important drivers of endemic freshwater fishes richness in Iran.

Predictor	Slope	*z*‐Value	*p*‐Value	AIC	D2
Intercept	−0.0906	−1.656.	>.05		
Annual mean temperature	0.92310	0.709	>.05	3754	0.004
Annual mean precipitation	10.68488	8.219***	<.000	2717	0.375
Temperature change velocity (LGM)	4.19334	4.082***	<.000	3721	0.016
Precipitation change velocity (LGM)	−9.85701	−4.492***	<.000	3080	0.245
Temperature change velocity (LIG)	−1.56268	−1.184	>.05	3601	0.059
Precipitation change velocity (LIG)	−4.01509	−2.082*	<.05	3503	0.094
Full model				2303	0.526

*Note*: The table shows estimates, associated *z*‐values, *p*‐values, the Akaike information criterion values (AIC), and explained deviance (D2) of variables.

### 3.3 Ecoregions similarity

**FIGURE 5:**
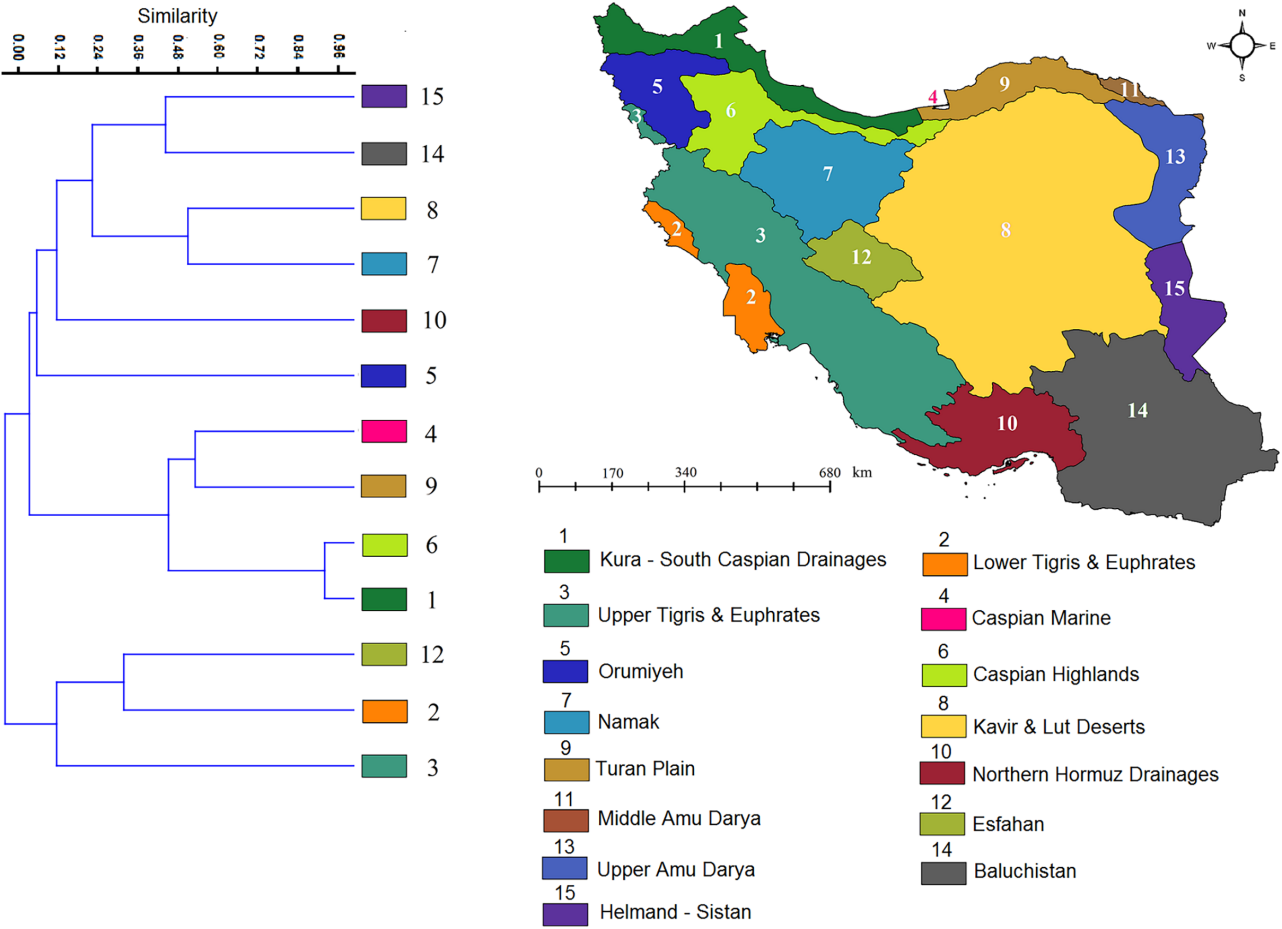
Dendrogram of the overall similarity for the 85 endemic freshwater fishes of Iran among the 15 ecoregions. Freshwater ecoregions of Iran (Abell et al., 2008). 1. Kura – South Caspian Drainages, 2. Lower Tigris & Euphrates, 3. Upper Tigris & Euphrates, 4. Caspian Marine, 5. Orumiyeh, 6. Caspian Highlands, 7. Namak, 8. Kavir & Lut Deserts, 9. Turan Plain, 10. Northern Hormuz Drainages, 11. Middle Amu Darya, 12. Esfahan, 13. Upper Amu Darya, 14. Baluchistan, 15. Helmand – Sistan.

Upper Tigris & Euphrates, followed by Northern Hormuz drainages were the most species‐rich ecoregions in Iran, with 53 and 14 species, respectively (Table 4). No endemic species was recorded in the Middle Amu Darya and Upper Amu Darya ecoregions. According to the dendrogram of similarity of the 85 endemic freshwater fishes of Iran among the 15 ecoregions, Orumiyeh was the most unique ecoregion according to endemic fish presence (Figure 5). Kura – South Caspian Drainages and Caspian Highlands were the most similar ecoregions based on the distribution of endemic freshwater fishes. Upper Tigris and Euphrates and Helmand–Sistan were the most dissimilar ecoregions in the country (Figure 5).

**TABLE 4:** Area and number of endemic freshwater fish species recorded in each freshwater ecoregion in Iran.

Ecoregions	Number of species
Kura – South Caspian Drainages	6
Lower Tigris & Euphrates	5
Upper Tigris & Euphrates	53
Caspian Marine	3
Orumiyeh	3
Caspian Highlands	5
Namak	9
Kavir & Lut Deserts	10
Turan Plain	4
Northern Hormuz Drainages	14
Esfahan	8
Baluchistan	6
Helmand – Sistan	1

## 4 DISCUSSION

Iran is a biodiversity‐rich country in the Middle East, and it is home to over 1317 vertebrate species (Yousefi et al., 2023) of which many are endemic to the country (Jouladeh‐Roudbar et al., 2020; Kafash et al., 2020). This study is the first to map endemic freshwater fish richness and determine the drivers of their richness pattern in Iran, a rather neglected and biodiversity‐unknown country in the Middle East.

Our results showed that Upper Tigris & Euphrates ecoregion harbors the highest number of endemic species; this makes the ecoregion a hotspot of freshwater biodiversity in Iran. This ecoregion geographically overlapped with the Zagros Mountains, which is known as an important biodiversity hotspot and center of origin for terrestrial vertebrates of Iran, like reptiles and mammals (Ahmadzadeh et al., 2013; Anderson, 1999; Hosseinzadeh et al., 2014; Kafash et al., 2020, 2021; Malekoutian et al., 2020; Vaissi, 2021; Yousefi et al., 2022). Previous studies have shown that the Zagros Mountains are a hotspot of terrestrial vertebrates in Iran (Hosseinzadeh et al., 2014; Kafash et al., 2020, 2021; Yousefi et al., 2022, 2023). In this study, we found that these mountains are also a hotspot for freshwater fishes in the country. Apart from being a hotspot of vertebrates, the Zagros Mountains served as important past climatic refugia for several groups of vertebrates (Ahmadzadeh et al., 2013; Farasat et al., 2016; Malekoutian et al., 2020; Perktaş et al., 2011; Vaissi, 2021). All of these make the mountains a critical geographic unit for the conservation of biodiversity in Iran.

Although we initially expected past climate to be the most influential factor in shaping freshwater fish richness in Iran, we found that current precipitation was the most crucial variable in explaining endemic freshwater fish richness pattern. Considering that fish are aquatic species positive correlation of fish richness with precipitation can be justified that areas that receive higher precipitation can provide optimal environmental conditions for them. Also, rainfall, floods, and a decrease in water temperature trigger spawning and migration in fishes. Similar to our study, several studies of other taxonomic groups also detected a stronger correlation between precipitation with species richness. For instance, precipitation was identified as the most important predictor of bat richness in Iran (Kafash et al., 2021). Precipitation change velocity since the LGM was identified as the second most important predictor of endemic freshwater fish richness. This is in line with previous studies which documented the role of past climate change in different fish species as well as species richness patterns in Iran (Gholami et al., 2015; Schwarzer et al., 2017).

The impacts of past climate change are well documented on the distribution of biodiversity across the globe (Hewitt, 2004; Jansson, 2003; Sandel et al., 2011; Theodoridis et al., 2020; Weigelt et al., 2016). Generally, areas that experienced higher climate change host fewer species and climatically stable areas harbor hotspots of species diversity and endemism (Brown et al., 2020; Harrison & Noss, 2017; Hewitt, 2004; Jansson, 2003; Sandel et al., 2011). In line with this general pattern and our initial expectation, we found a strong negative correlation between endemic freshwater fish richness and precipitation change velocity. This means that areas that were climatically less stable since the LGM host fewer endemic freshwater fish species.

Although in this study we did not consider the impact of mountain uplifting on the existing richness pattern of freshwater fishes in Iran, recent studies, have demonstrated a clear link between the diversification of these fishes and the uplift of the Zagros and Alborz mountains (Yousefi et al., 2023). For instance, Gholami et al. (2014) associated the diversification of the *Esmaeilius* genus with tectonic events during the Middle to Late Miocene period. Additionally, research by Ghanavi et al. (2016) has documented the formation of new lineages in the *Capoeta* genus in Iran due to the Zagros and Alborz uplifting. Therefore, it is evident that both climatic oscillations and mountain uplifting have played pivotal roles in shaping the current richness pattern of endemic freshwater fish species in Iran.

We estimated the similarity of freshwater ecoregions based on the endemic fish presence and absence. Estimated similarity among the ecoregions can be explained by past and current hydrological connectivity of the ecoregions (Esmaeili et al., 2020; Filipe et al., 2009; Gholami et al., 2014) which facilitated dispersal and faunal exchange among the ecoregions. Orumiyeh ecoregion was a unique ecoregion because 80% of its assemblage cannot be found in other ecoregions, thus it should be prioritized for conservation. There are clear biogeographic affinities between Orumiyeh ecoregion and the Aras adjacent to the north, even though it might only be 0.5 million years old. During past, humid climatic periods, probably the last time during the peak of the last glaciation, Urmia was a freshwater lake of a much bigger size allowing fishes to migrate from of tributary of the lake to another.

Freshwater ecosystems are at greater risk of biodiversity losses compared to their surrounding terrestrial ecosystems (Albert et al., 2021; Turak et al., 2017). But they are poorly protected globally meaning that they are more vulnerable to human activities (Szabolcs et al., 2022). Like other parts of the world, the freshwater ecosystems of Iran are poorly protected (Darvishsefat, 2008). This study contributed to the protection of freshwater biodiversity by identifying areas with the highest endemic freshwater fish richness. These areas should be included in the current protected areas of the country or considered for the selection of new protected areas to increase freshwater ecosystems' legal protection. This is particularly important because protected areas of Iran were initially designed to protect large mammals like wild goat (*Capra aegagrus*), wild sheep (*Ovis orientalis* and *Ovis vignei*), and Persian gazelle (*Gazella subgutturosa*) thus other vertebrate groups especially freshwater fishes were completely ignored (Darvishsefat, 2008).

The richness map created in this study is based on the current distribution records of endemic freshwater fishes of Iran. However, there are several factors that can change the identified richness pattern in the country, like climate change, land use change, invasive species, and habitat fragmentation through dam construction (Jouladeh‐Roudbar et al., 2020; Makki et al., 2021). For instance, it is predicted that endemic freshwater fish of Iran will shift their distribution in response to climate change (Makki et al., 2021; Yousefi et al., 2020). Thus, this map can serve as a baseline to document the role of environmental changes and human activities in altering biodiversity distribution patterns in general and endemic freshwater fish richness pattern in particular.

## 5 CONCLUSIONS

Freshwater fishes were the subject of numerous diversity mapping studies at a different spatial scale and geographical regions (Bogotá‐Gregory et al., 2020; Filipe et al., 2009; Hoeinghaus et al., 2007; Qian et al., 2021). But, freshwater fishes of Iran received no attention in this regard. Here, we presented the first richness map of all endemic freshwater fishes of Iran and showed that their richness is highest in the Zagros Mountains/ Upper Tigris & Euphrates ecoregion. This highlights that Zagros Mountains are an unquestionable biodiversity hotspot for all vertebrate groups in Iran. Advancing our knowledge on the spatial distribution of freshwater biodiversity in Iran, this study revealed that endemic freshwater fish richness is shaped by current and past climate. Future studies should examine the richness pattern of all freshwater fishes of the country to find a better understanding of spatial distribution of biodiversity in general and freshwater species in particular in Iran.

## AUTHOR CONTRIBUTIONS

**Masoud Yousefi:** Conceptualization (lead); formal analysis (lead); investigation (equal); methodology (equal); writing – original draft (lead); writing – review and editing (lead). **Arash Jouladeh‐Roudbar:** Formal analysis (equal); investigation (lead); methodology (equal); writing – original draft (supporting); writing – review and editing (equal). **Anooshe Kafash:** Formal analysis (equal); investigation (equal); methodology (lead); software (lead); writing – original draft (supporting); writing – review and editing (equal).

## CONFLICT OF INTEREST STATEMENT

The authors declare no conflicts of interest regarding this article.

## Supporting information


Appendix S1.

## Data Availability

All data used in this study are publicly available from the sources described in the method section and the supplementary material of this article. Current climate data are accessible form WorldClim (https://www.worldclim.org/data/worldclim21.html) and past climate data from PaleoClim (https://doi.org/10.1038/sdata.2018.254), other data are available at Kafash (2023) https://doi.org/10.5281/zenodo.10298559, and Yousefi et al. (2024) https://doi.org/10.5281/zenodo.10298559.
